# On the receiving end: have patient perceptions of the side-effects of cancer chemotherapy changed since the twentieth century?

**DOI:** 10.1007/s00520-022-06804-1

**Published:** 2022-01-11

**Authors:** Janette L. Vardy, Andre Liew, Anne Warby, Alexander Elder, Itay Keshet, Rhonda Devine, Calina Ouliaris, Corrinne Renton, Martin H. N. Tattersall, Haryana M. Dhillon

**Affiliations:** 1 Faculty of Medicine and Health, University of Sydney, Sydney, Australia; 2 Concord Cancer Centre, Concord Repatriation General Hospital, Hospital Rd., Concord, NSW 2137 Australia; 3 Centre for Medical Psychology and Evidence-based Decision-making, University of Sydney, Sydney, Australia; 4 School of Psychology, Faculty of Science, The University of Sydney, Sydney, Australia

**Keywords:** Symptoms, Chemotherapy, Chemotherapy side-effects, Patient-reported outcomes

## Abstract

**Background:**

Studies in 1983 and 1993 identified and ranked symptoms experienced by cancer patients receiving chemotherapy. We repeated the studies to obtain updated information on patient perceptions of chemotherapy-associated symptoms.

**Patients and methods:**

A cross-sectional interview and patient-reported outcome questionnaires were administered to out-patients receiving chemotherapy. Patients selected from 124 cards to identify and rank the severity of physical and non-physical symptoms they had experienced and attributed to chemotherapy (primary endpoint). The patient’s medical oncologist and primary chemotherapy nurse were invited to rank the five symptoms they believed the patient would rank as their most severe. We analysed the association of symptoms and their severity with patient demographics, chemotherapy regimen, and patient-reported outcomes. Results were compared to the earlier studies.

**Results:**

Overall, 302 patients completed the interview: median age 58 years (range 17–85); 56% female; main tumour types colorectal 81 (27%), breast 67 (22%), lung 49 (16%); 45% treated with curative intent. Most common symptoms (reported by >50%) were: alopecia, general weakness, effects on family/partner, loss of taste, nausea, fatigue, difficulty sleeping, effects on work/home duties, and having to put life on hold. The most severe symptoms (ranked by >15% in top five) were: concern about effects on family/partner, nausea, fear of the future, fatigue, not knowing what will happen, putting my life on hold, and general weakness. Perceptions of doctors and nurses of patients’ symptom severity closely matched patients’ rankings.

**Conclusions:**

Compared to earlier studies, there was an increase in non-physical concerns such as effects on family and future, and a decrease in physical symptoms, particularly vomiting, but nausea, fatigue and general weakness remained bothersome.

**Highlights:**

• Symptoms related to chemotherapy have changed over time, likely due to less toxic regimens and improvements in supportive care.

• Effects on family/partner, fear of the future, not knowing what will happen, and “life on hold” were major issues for patients.

• Vomiting has decreased but nausea, fatigue and general weakness remain common symptoms for chemotherapy patients.

**Supplementary Information:**

The online version contains supplementary material available at 10.1007/s00520-022-06804-1.

## Background

Two studies published in 1983 [1] and 1993 [2] identified and ranked symptoms experienced by patients with cancer who were receiving chemotherapy. In 1983, 99 patients who were receiving chemotherapy reported that non-physical side effects constituted 54% of the 15 most severe symptoms; these included the thought of coming for treatment, length of time treatment takes, and having to have a needle. Major physical side effects were vomiting, nausea and hair loss. When physical and non-physical categories were combined, vomiting, nausea and hair loss remained the most severe [1].

A decade later, patients reported a reduction in the severity of some symptoms, particularly vomiting, and a shift from concerns about physical to psychosocial issues. Nausea was the most severe symptom followed by tiredness and alopecia. Vomiting was of lesser concern, reflecting the introduction of 5HT3 antagonist anti-emetics. Concern about the effect on friends and family increased in rankings from 10^th^ to 3^rd^. In both studies, differences were seen in the symptoms experienced and their severity, based on chemotherapy regimen, age, and sex [2].

Similar methodology was applied to 100 French patients with advanced cancer, recruited between 1998 and 2000 [3]. The side-effect identified as most common and severe was “affects my family or partner”, followed by “loss of hair”, then “constantly tired”.

Since 2000, there have been major changes in chemotherapy and in supportive care to manage side-effects and symptoms, increased patient involvement in cancer care decisions, and people wanting more information about their treatment including side-effects [4–6]. Here, we provide information on patient perceptions of chemotherapy-associated symptoms in people being treated with modern chemotherapy and supportive care including anti-emetic regimens.

## Methods

This cross-sectional survey was conducted as a face-to-face interview with patient-reported outcome measures (PROM) on one occasion when patients were attending out-patient medical oncology clinics at two metropolitan teaching hospitals (one of which was included in the prior studies) and one regional hospital, between May 2008 and October 2016. Patients with any stage cancer, who were receiving chemotherapy, were invited to participate in the study. Eligibility criteria included a diagnosis of invasive cancer, currently receiving chemotherapy for a solid malignancy and completion of at least one cycle, and sufficient English to complete PROM.

### Procedures

The methodology used in the previous studies [1, 2], with additional questions, was retained to maximise validity of comparisons. Additional symptoms were added to the original list by an expert group that consisted of two medical oncologists, a senior cancer nurse, social worker, clinical psychologist, and behavioural scientist. The additional items (highlighted in Table 1) were piloted on 10 patients to assess understanding and construct validity.

**Table 1:** List of possible symptoms associated with chemotherapy

Group A — physical symptoms		
Acne (pimples)	Heart beating faster	Painful/tender veins around or above the injection site^
Ankle or legs swelling (retaining water)^	Hiccups^	Passing more water than usual (increased urination)
Being sick (vomiting)	Hot flushes	Peeling hands and/or feet^
Bruise easily	Increased appetite	Periods become irregular
Burning palms and/or soles of feet^	Increased hair growth on legs	Periods stop
Cannot taste things	Increased thirst	Pins and needles in fingers and toes
Change in the way things taste	Indigestion/reflux/discomfort^	Ringing in ears
Changes in how things smell	Itch^	Runny nose^
Changes in skin colour	Itching at injection site	Runny or watery eyes^
Coloured urine	Joint aches and pains^	Shaking all over
Constantly tired	Joint stiffness^	Shortness of breath
Cough^	Loss of appetite	Skin rash
Deafness	Loss of hair	Sore eyes^
Difficulty sleeping	Loss of liquid or frequent bowel action (diarrhoea)	Sore hands and/or feet^
Dry mouth^	Loss of weight	Sore mouth
Dry skin	Mouth sores (ulcers) ^	Sore throat
Fatigue (tiredness)	Nail changes^	Sore, tender muscles^
Feeling sick (nausea)		
Fever and/or chills^	Nosebleeds	Stuffy nose^
Fingernails go brown	Not having regular bowel action (constipation)	Swollen tummy (abdominal fullness)
General aches and pains	Numbness in fingers and toes	Thrush in your mouth^
General weakness^	Pain around fingernails^	Trouble with swallowing
Giddiness or dizziness on standing up	Pain passing water	Tummy ache (abdominal pain)
Headache, migraine	Pain when swallowing^	Weight gain
Group B — non-physical symptoms		
Cannot concentrate	Feeling of not coping generally with treatment	My cancer makes me different^
Cannot get clothes to fit	Feeling overwhelmed^	My life is on hold^
Constant reminder of my disease^	Feeling that the treatment is damaging my body	No end to treatment^
Cost of treatment^	Feeling unattractive^	Not being able to choose where you sit for treatment^
Crying more often	Forget things	Not getting preferred place in the chemo suite^
Dependence on others^	Frequency of treatment^	Not having the chance to ask the doctor questions
Difficulty finding words^	Getting started in the mornings^	Not knowing what will happen^
Effects my family or partner	Having to come to the clinic rather than a private doctor	Not seeing the same doctor each time
Effects my home/work duties	Having to have a needle	Not seeing the same nurses/staff each time^
Effects my social activities	Having to wait for treatment with other patients	Not understanding what is happening
Excessive time waiting for chemo^	Infertility (cannot have children)	People looking at me^
Fear of the future^	Lack of choice of appointment times^	Seeing very sick people^
Feeling angry	Lack of privacy in the chemo suite^	Slow thinking (fuzzy head) ^
Feeling anxious, tense or worried^#^	Length of time treatment takes at clinic	Thought of coming for treatment
Feeling bad tempered (irritability)	Loss of independence^	Trouble finding somewhere to park
Feeling like emotions are out of control^	Loss of sexual ability	Trouble getting to the clinic
Feeling low, miserable (depression)	Loss of sexual feeling	Unwanted advice^
Feeling of having to have treatment which I don't think will do any good	Money worries^	Worried about my job^
Feeling of having to have treatment which I don't want		

^ Additional symptoms added that were not in the original 1983 or 1993 studies

^#^Modified wording from original adding “or worried”

The patient interview was scripted and was conducted by trained research assistants. The procedure was as described in the previous two studies [1, 2]. In brief, two sets of white cards were prepared. Group A listed physical side-effects of chemotherapy (*n* = 69) and Group B listed non-physical side-effects of chemotherapy (*n* = 55) (Table 1). Both card sets were shuffled, and Group A cards were presented first, one at a time. The participant was asked to select all cards that described a symptom they had experienced and that they attributed to their current chemotherapy. They were then asked to rank the selected cards in order of severity. This process was repeated for Group B cards. The five highest ranked cards from each group were combined and the patient was asked to select and rank the five most severe symptoms regardless of group. Five points were allocated to symptoms ranked as the most severe, decreasing to one point for symptoms ranked as least severe. The allocated points were used to generate an overall ranking of symptom severity. The patient’s medical oncologist and a nurse closely involved in their care were also asked to rank the five symptoms they believed that each patient would rank as their most severe. They were given a separate list of physical and non-physical symptoms, grouped by systems or domains, and asked firstly to rank the top 5 for each list. They were then asked to rank the top 5 across both symptom lists.

Demographic, disease, and treatment characteristics were collected from the participant and their medical record. The research assistant rated the participant’s performance status based on European Co-operative Oncology Group (ECOG) criteria [7], and determined their Colinet Simplified Comorbidity Score [8].

An additional component done after the patient interview was completion of several health-related quality of life PROMs. These included self-rating of ECOG performance status [7], quality of life (QOL) and fatigue assessed by the Functional Assessment of Cancer Therapy – General (FACT-G) and Fatigue (FACT-F) subscale [9, 10], anxiety and depression assessed by the 12-item General Health Questionnaire (GHQ-12) [11], and the EQ-5D Thermometer Scale of overall health state [12]. In addition, patients retrospectively completed linear analogue self-assessment (LASA) scales documenting the severity of anticipatory nausea and vomiting, and nausea and vomiting within and more than 24 h after chemotherapy for their last cycle of chemotherapy.

Ethical approval was granted by each hospital and all participants provided written, informed consent.

### Statistical analysis

The 1983 and 1993 studies had 99 and 150 patients respectively. We increased the planned sample size to 400 patients to increase the generalisability of the study and to determine if there was a difference across the number of chemotherapy cycles. Statistical analyses were conducted using Stata 13.

The primary aim was to determine the most severe symptoms experienced by cancer patients while receiving chemotherapy. Descriptive statistics were used to describe the frequency of symptoms and to rank their severity. Major secondary goals included: (i) comparison of symptoms with those reported in the studies conducted in 1983 and 1993; (ii) the association of symptoms with patient characteristics such as sex, age, chemotherapy regimen (platinum-based, taxane, and other), number of prior chemotherapy cycles and PROMs.; (iii) comparison of patients’ ratings of symptom severity with that of their physicians and nurses.

For the LASA scales, scores ranged from 0 (no discomfort) to 100 (extreme discomfort). Consistent with the 1993 study, a score of >75 was interpreted as indicating a substantial level of discomfort. Proportions were reported.

Logistic regression models were used to analyse the association of symptoms and their severity with patient demographics, chemotherapy regimen and PROMs, except for PROMs measured on a continuous scale when linear regression models were used. The regression models used the demographic, treatment or PROM variable as the dependent variable and each of the symptoms as exploratory/independent variables. Consistent with the previous studies, we restricted the analysis to comparing whether each symptom was included among the five most severe.

The 1983 study included only patients undergoing treatment for advanced cancer, whereas the 1993 study included people with earlier stage disease, so for comparison between studies we restricted analysis only to those with advanced/metastatic cancer. Raw data were not available from the previous studies, so we compared severity rankings for symptoms.

## Results

The study was completed over two time periods from 2008 to 2013 (*n* = 272 patients) and from January to October 2016 (*n* = 30) due to limitations in resources.

**Fig. 1:**
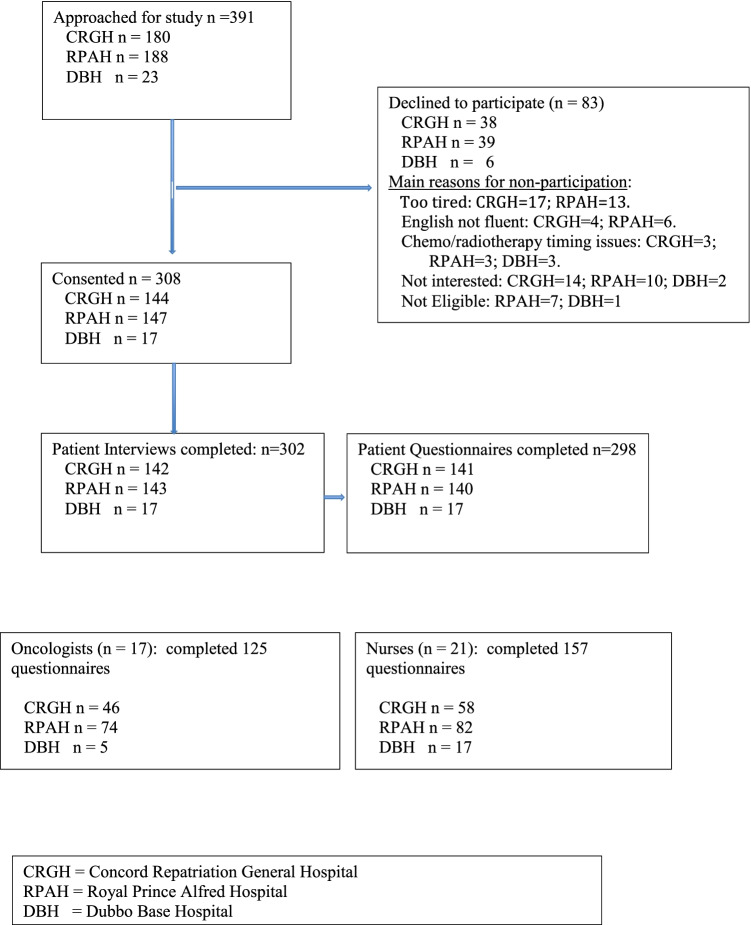
Consort diagram

Of 391 patients approached, 308 consented to participate and 302 completed the assessment (Fig. 1, Consort diagram). Reasons for declining to participate were: felt too unwell; lack of interest; insufficient English; and unable to schedule.

The median age of participants was 58 years (range 17–85) and 56% were female (Table 2). The predominant tumour types were colorectal (27%), breast (22%), and lung (16%). Forty-five percent were being treated with curative intent. The median time since diagnosis was 6 months (range 1–392). In total, 54% of patients were receiving a platinum-based chemotherapy regimen, and 31% a taxane-containing regimen. The median number of cycles received prior to the interview was 4 (range 1–23).

**Table 2:** Demographic and disease characteristics of the sample (*N* = 302)

Variable	
Age (yrs)	*N* = 302
Median (IQR) (Range)	58 (17) (17–85)
Months from diagnosis	*N* = 300
Median (IQR) (Range)	6 (22) (1–392)
Female, n (%)	168 (56)
Marital status, n (%)	*N* = 302
Married/defacto	210 (70)
Separated/divorced	37 (12)
Single	38 (13)
Widowed	17 (6)
Living arrangement, n (%)	*N* = 302
Alone	58 (19)
With others	244 (81)
Country of birth, n (%)	
Australia	198 (66)
Other	104 (34)
Primary language, n (%)	*N* = 302
English	258 (85)
Non-English speaking	44 (15)
Education level completed, n (%)	*N* = 301
Primary school	14 (5)
Secondary school	129 (43)
College/University	111 (37)
Post-graduate	47 (16)
Work status, n (%)	*N* = 302
Full-time employment	151 (50)
Part-time employment	42 (14)
Home duties	9 (3)
Retired	91 (30)
Unemployed	9 (3)
Primary site of cancer, n (%)	*N* = 302
Colorectal	81 (27)
Breast	67 (22)
Lung	49 (16)
Gynaecological	30 (10)
Genitourinary	16 (5)
Upper gastrointestinal	13 (4)
Other	46 (15)
Stage, n (%)	*N* = 295
I	10 (3)
II	25 (8)
III	100 (34)
IV	160 (54)
ECOG (patient reported), n (%)	*N* = 300
0	52 (17)
1	198 (66)
2	40 (13)
3	9 (3)
4	1 (0)
Other cancer treatments received, n (%)	*N* = 291–299
Surgery	214/299 (72)
Radiotherapy	76/299 (25)
Endocrine	29/292 (10)
Other anticancer treatment(s)	21/291 (7)
Current chemotherapy, n (%)	*N* = 296
Adjuvant	111 (38)
Neo-adjuvant	20 (7)
Metastatic	132 (45)
Palliative	19 (6)
Other	14 (5)
Current chemotherapy regimen, n (%)	*N* = 296
Platinum-based	158 (54)
Taxane	91 (31)
Chemotherapy and targeted therapy	17 (6)
Current Chemotherapy cycle number	*N* = 291
Median (IQR) (Range)	4 (3) (1–23)
No. of lines of chemotherapy	*N* = 296
Median (IQR) (Range)	1 (4) (1–8)

### Symptoms experienced and severity

Patients reported experiencing a median of 29 symptoms: 18 physical and 11 non-physical. The most common symptoms were loss of hair, general weakness, concern about effects on family or partner, loss of taste, nausea, fatigue, difficulty sleeping, concern about effects on work or home duties, and having to put life on hold. These were reported by >50% of patients. (Table 3, Supplementary Tables 1 and 2). While other symptoms were less commonly reported, 58 of 124 symptoms were reported by >25% of patients.

**Table 3:** Frequency and severity of symptoms experienced by patients and comparison with severity ratings of doctors and nurses

Rank	Symptom frequency (Patients)	% Patients reporting	Symptom severity (Patients)	Symptom severity (Doctors)	Symptom severity (Nurses)
	*N* = 302		*N* = 302	*N* = 96	*N* = 115
1	Loss of hair	67.9	Effects my family or partner	Fatigue	Fatigue
2	General weakness	65.9	Nausea	Nausea	Nausea
3	Effects my family or partner	65.6	Fear of the future	Effects my family or partner	General Weakness
4	Change in the way things taste	58.3	Fatigue	Not knowing what will happen	Effects my family or partner
5	Nausea	57.9	Not knowing what will happen	Vomiting	Alopecia
6	Fatigue	55.6	My life is on hold	General Weakness	Excessive time waiting for chemo
7	Difficulty sleeping	52.3	General weakness	Fear of the future	Vomiting
8	Effects my work/home duties	51.4	Difficulty sleeping	Length of time treatment takes at clinic	Fear of the future
9	My life is on hold	50.3	Loss of hair	Loss of appetite	Dependence on others
10	Dry mouth	49.7	Constipation	Constant reminder of my disease	Having to have a needle

### Association of symptoms with patient characteristics

Differences associated with the chemotherapy regimen, tumour type and with number of cycles were most often physical symptoms (Supplementary Tables 3 and 4). Patients on platinum-based regimens were more likely to report cough and pain when swallowing, with pins and needles, general weakness, loss of appetite and indigestion rated as more severe compared with other regimens (Supplementary Table 3 and 4). Patients receiving taxanes were more likely to report loss of hair (and to rate it as more severe), general aches and pains, and skin rash. There were no significant differences across drug regimens in the non-physical symptoms regarded as most severe.

Patients who had received more cycles of chemotherapy were more likely to report pins and needles, diarrhoea, abdominal pain, and thrush, with headaches and sore/tender muscles being rated more severe. They were also more likely to report feeling their cancer made them different. In contrast, they were less likely to report symptoms like indigestion, joint aches and pains, and ringing ears.

Women were more likely than men to report many physical and non-physical symptoms including loss of hair, pins and needles, headache, slow thinking, crying more often, and feeling unattractive. The only symptoms reported more frequently by men were passing more urine and hiccups, with trouble swallowing rated as more severe. Younger patients (under 60 years) were more likely to report concerns about the effects on work or home duties as well as difficulty sleeping and feeling angry. Older patients were more likely to report shortness of breath and easy bruising, with general weakness, changes in taste and constipation rated as more severe.

Patients treated with curative intent were more likely than those with advanced disease to report physical symptoms such as hot flushes, changes in smell, and sore eyes as well as non-physical symptoms like crying more often, seeing very sick people, and feeling of having to have treatment I don’t want (Supplementary Table 3). They were also more likely to rate as severe alopecia and feeling treatment is damaging (Supplementary Table 4). Patients treated with palliative intent were more likely to report loss of appetite, abdominal pain, cough, and feeling that cancer makes me different, no end to treatment and not understanding what is happening.

The most severe symptom was concern about the effects on family or partner, with 34% of patients ranking it among their five most severe symptoms and 12% as the most severe (Table 3). The next most severe symptoms were nausea, fear of the future, fatigue, not knowing what will happen, having to put my life on hold, and general weakness; these were ranked by >15% of patients among their five most severe symptoms.

**Table 4:** Symptom severity associated with quality of life, depression and anxiety, fatigue, self-reported performance status, and overall health using linear regression models

Patient-reported outcome measure	Symptom (from interview)	Change in variable (95% CI)	p-value
Quality of life:	My life is on hold	−5.8 (−10.7–−0.9)	0.02*
FACT-G	Depression	−9.3 (−18.1–−0.6)	0.04*
	Feeling unattractive	−25.1 (−43.0–−7.3)	0.01**
	Cost of treatment	−24.1 (−42.5–−5.8)	0.01*
Depression and anxiety	Depression	4.7 (1.6–7.9)	0.004**
GHQ-12	Feeling overwhelmed	5.1 (1.2–8.9)	0.01
	Cost of treatment	6.8 (0.0–13.5)	0.05*
Fatigue	Fatigue	−6.7 (−10.0–−3.3)	<0.001**
FACT-F	Cost of treatment	−14.8 (−29.6–−0.1)	0.05*
Self-reported performance status (ECOG)	Fatigue	4.1 (1.3–13.2)	0.02*
	My life is on hold	4.1 (1.4–12.2)	0.01*
Overall health	Nausea	−6.7 (−12.8–−0.6)	0.03*
Thermometer scale	Effects home/work duties	−9.8 (−18.2–−1.4)	0.02*
	My life is on hold	−9.7 (−16.1–−3.4)	0.003**

*p < 0.05

**p < 0.01

*FACT*, Functional Assessment of Cancer Treatment; *G*, general; *F*, fatigue subscale.

*GHQ*, General Health Questionnaire 12

*ECOG*, European Cooperative Oncology Group

Thermometer Scale = EQ-5D Thermometer Scale of overall health state

Severity of several non-physical symptoms, including having to put life on hold and cost of treatment, were associated with lower overall quality of life as measured by FACT-G and higher levels of depression and anxiety as measured by GHQ-12 (Table 4). Having to put life on hold and severe fatigue were associated with worse self-reported ECOG performance status. Other PROMS reflected well the patient reported symptoms.

**Table 5:** Percentage of patients with severe nausea and vomiting in relationship to timing of chemotherapy based on linear analogue self-assessment scale (LASA) rating >75/100: for all participants, and by chemotherapy regimen

Nausea and vomiting	Overall	Platinum-based	Taxane
	*N* = 298	*N* = 151	*N* = 88
Vomiting: pre chemotherapy	0.0	0.0	0.0
Nausea: pre chemotherapy	1.3	1.3	2.2
Vomiting: within 24 h	4.4	5.3	2.2
Nausea: within 24 h	8.4	7.9	6.7
Vomiting: 24 h post chemotherapy	3.7	5.9	2.2
Nausea: 24 h post chemo	9.7	11.2	5.6

### Comparison of patients’ symptom severity with that of doctors and nurses

Symptoms ratings by doctors and nurses were available for 96 and 116 patients respectively: their ranking of symptom severity was similar to the full sample. Perceptions of doctors and nurses matched closely the patients’ own rankings (Table 3). Six of the 10 symptoms ranked most severe by patients were also ranked by doctors and nurses among the 10 most severe. Having to put life on hold, difficulty sleeping, and constipation were ranked more highly by patients than doctors or nurses. Length of time spent at clinic and in waiting for chemotherapy, vomiting, and having to have a needle were ranked as more severe by doctors and nurses than by patients.

### Changes in symptom severity since 1983 and 1993 in people with advanced cancer

Vomiting and nausea declined in the severity ranking across study years. Vomiting, ranked as the most severe symptom in 1983 and fifth most severe in 1993, was ranked 23^rd^ among symptoms of patients with advanced disease in the current study (Supplementary Table 5). Nausea, ranked as the most severe symptom in 1993, was ranked as the fifth in the current study. Conversely, concerns about effects on family increased in severity (ranked 10th in 1983, fourth in 1993 and first in the current study). Depression decreased in severity, falling to 51st in the current study while the ranking of anxiety remained more stable. Some of the non-physical symptoms that ranked highly in terms of severity in the current study, such as fear of the future, and feeling like my life is on hold, were not surveyed in the previous studies.

### Nausea and vomiting pre, within and post 24-h of chemotherapy

Only 1% of patients in the current study had substantial anticipatory nausea (score of >75), and no patient reported substantial anticipatory vomiting compared with 17% and 5% in the previous studies (Table 5). Within 24 h of chemotherapy, 8% of patients reported substantial nausea and 4% substantial vomiting compared with 51% and 24% previously. Beyond 24 h after chemotherapy, 10% reported severe nausea and 4% severe vomiting compared with 57% and 29% in the previous studies. The results from LASA scales are consistent with the symptom ranking and indicate that the severity of nausea and vomiting has declined substantially since 1983.

Vomiting during the 24 h after chemotherapy and delayed vomiting after 24 h were strongly associated with higher ranking of vomiting whereas anticipatory vomiting was not. In contrast, anticipatory nausea, in addition to nausea within 24 h and after 24 h of chemotherapy, was a strong predictor of patients’ nausea ranking.

## Discussion

It is almost 40 years since the original ‘On the Receiving End’ study was published. In 1983, the top five severe symptoms were: vomiting, nausea, loss of hair, thought of coming for treatment, and length of time treatment takes, which was especially a concern for men. With improvement in management of acute physical symptoms, particularly vomiting, there has been a shift to patients reporting greater concern with non-physical side effects, particularly the impact of their cancer and/or treatment on their partner and family. In the current study, this was rated in the top five most severe symptoms by 34% and as the most severe by 12% of participants. By comparison, it was tenth in severity in 1983. Other highly rated symptoms in our study included fear of the future, uncertainty of what will happen and having to put their life on hold, which were not options in the earlier studies.

While major advances have been made in the prevention and treatment of vomiting, nausea remains difficult to manage and debilitating for patients [13]. Fatigue also continues to be a major problem with 56% of patients reporting fatigue and 66% general weakness. Our longitudinal study in colorectal cancer patients found that 70% of patients reported fatigue immediately following adjuvant chemotherapy compared with 31% who had surgery alone and 22% of healthy controls [14], with 44% still reporting fatigue 6 months after chemotherapy compared to ~30% of non-chemotherapy patients and controls. Studies in women with breast cancer have reported similar results [15, 16].

In the present study, 45% of patients were being treated with curative intent, most with a platinum or taxane regimen, and all patients were treated in the outpatient department. In the original 1983 study, all participants had advanced cancer and many required treatment administration as inpatients. Differences were more likely to be in physical symptoms across chemotherapy regimens rather than non-physical symptoms. By comparison, the 1993 study included a third of patients being treated with adjuvant chemotherapy.

Patients are generally better prepared for physical symptoms with chemotherapy than they are for psychosocial issues. This is likely because many health care professionals focus more on physical side-effects when providing information and obtaining informed consent prior to chemotherapy [17] and in eliciting symptoms in subsequent consultations [18, 19]. With improved physical outcomes, fear of cancer recurrence or progression, and psychosocial issues have become more salient. The similarities in non-physical symptoms ranked as severe across treatment regimens suggests the commonality of these concerns across tumour types and a need to address them more effectively. Results of the present study, and other studies showing cancer survivors have high psychological distress [20], fear of cancer recurrence, increased use of health services and poorer quality of life [21], suggest that mental health and well-being in cancer patients require greater attention.

Detecting and monitoring of symptoms experienced by patients receiving chemotherapy is an essential component of quality care. Changes in symptom profile may necessitate modification to the treatment regimen, or the provision of additional supportive care and patient education. Several studies have shown that clinicians tend to underestimate the incidence and severity of symptoms when compared to patient self-report [22–24]. Clinician accuracy in detecting patient symptoms has been reported to be lower for more subjective symptoms (e.g. fatigue and dyspnoea) than for symptoms that can be observed directly (e.g. vomiting and diarrhoea) [24]. However, in the present study, the perceptions of the oncologists and cancer nurses in rating the top five symptoms were fairly consistent with patient self-report, with oncologist awareness of troublesome non-physical symptoms greater than expected.

### Limitations and strengths

Our study provides important updated information to the oncology community regarding the symptoms that patients undergoing chemotherapy experience. We acknowledge that this may be different to the symptoms they are most concerned about, and that financial concerns are likely to be much greater in countries that do not have universal health care. We did not meet our planned sample size of 400, but recruited ~300 participants covering a broad spectrum of tumour type, chemotherapy regimens and disease stage. All patients were currently receiving chemotherapy and we investigated a wide array of symptoms. Data collection was spread over several years and there may have been changes in treatment and supportive care during this time which impacted symptoms. Targeted therapies and immunotherapy are not captured in this study with the exception of 6% of participants receiving a targeted therapy (e.g., trastuzumab) together with chemotherapy.

We recognise that there is confounding between some variables (for example all breast cancer patients were female, and some chemotherapy regimens are used only for certain cancer types), which limits the analysis of symptoms as a function of type of cancer, age and sex. Our matched data between patients and staff was limited to ~100 patients. Although the ranking of symptom severity by patients was similar to the larger cohort, it is possible that there was self-selection of oncologists and nurses who were more likely to discuss concerns with their patients. If so, we may have over-estimated their appreciation of symptoms most important to patients, but we would like to think that this may be due to oncology staff increasingly engaging in more patient-centred practice.

## Conclusions

There has been a change in the symptoms that patients undergoing chemotherapy find most bothersome. While nausea, fatigue and general weakness remain common, the effect on family or partner was rated as the most severe, with fear of the future, not knowing what will happen, and putting my life on hold also major issues.

## Supplementary Information

ESM 1 (DOCX 61 kb)

## Data Availability

Data will be shared on reasonable request to the corresponding author.
