# Supplementary material for: On the receiving end: have patient perceptions of the side-effects of cancer chemotherapy changed since the twentieth century?
Source: Support Care Cancer. 2022 Jan 11;30(4):3503–12. doi: 10.1007/s00520-022-06804-1 (PMC8857157; doi:10.1007/s00520-022-06804-1)
Supplement: Supplementary file 1 — (DOCX 61 kb) [file 520_2022_6804_MOESM1_ESM.docx]

**Supplementary information**

Supplementary Table 1 Frequency and severity of symptoms experienced by patients: Group A – Physical Symptoms

| Symptom | Patients reporting symptom (%) | Overall severity ranking |
| --- | --- | --- |
| Loss of hair | 67.9 | 9 |
| General Weakness | 65.9 | 7 |
| Change in the way things taste | 58.3 | 13 |
| Feeling sick (nausea) | 57.9 | 2 |
| Fatigue | 55.6 | 4 |
| Difficulty sleeping | 52.3 | 8 |
| Dry mouth | 49.7 | 44 |
| Constipation | 48.7 | 10 |
| Loss of appetite | 48.0 | 26 |
| Indigestion/reflux/discomfort | 40.7 | 27 |
| Pins and needles fingers or toes | 39.7 | 22 |
| Loss of weight | 39.0 | 30 |
| Shortness of breath | 38.7 | 17 |
| Dry skin | 38.4 | 68 |
| Runny nose | 37.7 | 41 |
| Diarrhoea | 33.8 | 11 |
| General aches and pains | 33.4 | 56 |
| Giddiness or dizziness on standing up | 33.4 | 58 |
| Cannot taste things | 33.1 | 52 |
| Numbness in fingers or toes | 32.1 | 33 |
| Passing more water | 32.1 | 65 |
| Increased thirst | 30.5 | 74 |
| Joint aches and pains | 30.1 | 21 |
| Hot flushes | 29.5 | 54 |
| Increased appetite | 28.8 | 72 |
| Mouth sores (ulcers) | 28.8 | 34 |
| Sore, tender muscles | 28.5 | 51 |
| Changes in how things smell | 27.8 | 76 |
| Being sick (vomiting) | 27.5 | 15 |
| Weight gain | 26.5 | 23 |
| Coloured urine | 25.8 | 88 |
| Painful/tender injection site | 24.8 | 61 |
| Fever and/or chills | 24.5 | 84 |
| Nail changes | 23.5 | - |
| Runny or watery eyes | 23.5 | 36 |
| Bruise easily | 23.2 | 101 |
| Tummy ache (abdominal pain) | 22.5 | 29 |
| Swollen tummy | 22.2 | 40 |
| Headache, migraine | 21.9 | 63 |
| Cough | 21.2 | 46 |
| Sore mouth | 20.5 | 38 |
| Skin rash | 19.5 | 60 |
| Itch | 19.2 | 66 |
| Nose bleeds | 18.9 | 75 |
| Changes in skin colour | 18.9 | - |
| Sore eyes | 17.5 | 96 |
| Joint stiffness | 16.9 | 103 |
| Sore throat | 16.9 | 87 |
| Hiccups | 16.6 | 98 |
| Sore hands and/or feet | 16.2 | 73 |
| Ankle or legs swelling | 15.9 | 64 |
| Trouble with swallowing | 15.2 | 50 |
| Ringing in ears | 13.9 | 109 |
| Stuffy nose | 13.9 | 90 |
| Heart beating faster | 13.2 | 83 |
| Peeling hands and/or feet | 13.2 | 110 |
| Acne (pimples) | 11.3 | 106 |
| Pain when swallowing | 10.9 | 92 |
| Burning palms/soles of feet | 10.6 | 78 |
| Pain around fingernails | 10.3 | - |
| Periods stop | 8.6 | - |
| Thrush in your mouth | 8.6 | 100 |
| Deafness | 7.0 | 93 |
| Shaking all over | 7.0 | 80 |
| Itching at injection site | 6.6 | 108 |
| Fingernails go brown | 6.3 | - |
| Pain passing water | 6.0 | - |
| Periods become irregular | 5.0 | - |
| Pt has no symptoms | 3.3 | - |
| Increased hair growth on legs | 1.3 | - |

Supplementary Table 2 Frequency and severity of symptoms experienced by patients: Group B – Non-physical Symptoms

| Symptom | Patients reporting symptom (%) | Overall severity ranking |
| --- | --- | --- |
| Effects my family or partner | 65.6 | 1 |
| Effects my work/home duties | 51.4 | 21 |
| My life is on hold | 50.3 | 6 |
| Fear of the future | 49.0 | 3 |
| Effects my social activities | 48.0 | 25 |
| Feeling anxious or tense or worried | 48.0 | 16 |
| Not knowing what will happen | 46.9 | 5 |
| Slow thinking (fuzzy head) | 41.8 | 42 |
| Dependence on others | 41.5 | 12 |
| Forget things | 41.5 | 49 |
| Feeling low, miserable (depression) | 38.1 | 31 |
| Cannot concentrate | 37.8 | 57 |
| Thought of coming for treatment | 37.1 | 71 |
| Feeling bad tempered (irritability) | 36.4 | 53 |
| Feeling that the treatment is damaging my body | 33.3 | 14 |
| Constant reminder of my disease | 32.7 | 37 |
| Loss of independence | 32.3 | 28 |
| Loss of sexual feeling | 31.3 | 47 |
| Difficulty finding words | 31.0 | 67 |
| Feeling overwhelmed | 29.3 | 59 |
| Excessive time waiting for chemo | 27.9 | 32 |
| Length of time treatment takes at clinic | 27.9 | 48 |
| Getting started in the mornings | 27.2 | 39 |
| Having to have a needle | 27.2 | 19 |
| My cancer makes me different | 27.2 | 70 |
| Loss of sexual ability | 26.9 | 35 |
| Crying more often | 26.2 | 91 |
| Money worries | 24.8 | 18 |
| Feeling angry | 23.5 | 81 |
| No end to treatment | 23.1 | 24 |
| Trouble finding park | 21.8 | 45 |
| Feeling like emotions are out of control | 21.1 | 62 |
| Feeling unattractive | 20.1 | 104 |
| Seeing very sick people | 19.7 | 86 |
| Unwanted advice | 19.7 | 69 |
| Frequency of treatment | 17.7 | 86 |
| Worried about my job | 15.0 | 55 |
| Feeling of having to have treatment which I don't want | 13.6 | 77 |
| Feeling of not coping generally with treatment | 12.9 | - |
| Cannot get clothes to fit | 10.5 | - |
| People looking at me | 9.9 | 95 |
| Lack of choice of appointment times | 9.5 | 102 |
| Cost of treatment | 8.5 | 82 |
| Not understanding what happening | 8.2 | 79 |
| Feeling treatment won't do good | 8.2 | 94 |
| Infertility (cannot have children) | 7.1 | 43 |
| Not having the chance to ask the doctor questions | 6.1 | 99 |
| Not seeing the same doctor each time | 6.1 | 97 |
| Trouble getting to the clinic | 5.8 | 107 |
| Not seeing same nurses/staff | 4.4 | - |
| Having to wait for treatment with others | 4.1 | 105 |
| Lack of privacy in chemo suite | 3.4 | - |
| Having to come to the clinic rather than a private doctor | 1.7 | - |
| Not being able to choose where sit | 1.4 | - |
| Not getting preferred place in the chemo suite | 1.4 | - |

Supplementary Table 3 Percent (%) of patients reporting symptoms, which differentiated chemotherapy regimens, tumour types and demographic groupings using logistic regression models with odds ratio (OR) and 95% confidence intervals (95% CI)

| Symptom | %; OR, (95% CI) | %; OR, (95% CI) | %; OR, (95% CI) |
| --- | --- | --- | --- |
|  | Colorectal  (N=81) | Breast  (N=67) | Lung  (N=49) |
| Pins and needles | 63%; 20.4 (4.2-98.2)* | 39%; 2.6 (0.6-11.2) | 18%; 0.0 (0.0-0.2) |
| Diarrhoea | 48%; 4.6 (1.2-17.5)* | 45%; 9.9 (2.0-49.6)* | 14%; 0.0 (0.0-0.8) |
| Numbness | 47%; 7.3 (1.7-31.0)* | 34%; 0.7 (0.2-3.0) | 20%; 0.0 (0.0-0.9) |
| Frequency of treatment | 26%; 4.8 (1.7-13.6)* | 16%; 0.5 (0.2-1.4) | 8%; 0.3 (0.1-1.9) |
| Loss of hair | 46%; 0.1 (0.0-0.5) | 88%; 6.1 (1.0-36.1)* | 63%; 0.0 (0.0-0.6) |
| Difficulty sleeping | 41%; 0.7 (0.2-2.5) | 73%; 21.3 (3.6-127.6)* | 43%; 0.3 (0.0-4.2) |
| Nail changes | 30%; 0.6 (0.1-2.9) | 37%; 4.4 (1.0-18.7)* | 8%; 0.0 (0.0-0.1) |
| Runny or watery eyes | 16%; 0.4 (0.1-1.9) | 43%; 5.9 (1.1-30.9)* | 22%; 2.4 (0.1-126.8) |
| Headache | 17%; 0.2 (0.0-1.2) | 43%; 11.9 (2.8-49.4)* | 10%; 3.5 (0.0-5024.8) |
| Joint stiffness | 9%; 2.6 (0.3-22.7) | 34%; 25.7 (2.5-261.4)* | 14%; 0.0 (0.0-1.2) |
| Sore throat | 16%; 0.9 (0.2-5.3) | 30%; 13.3 (2.1-83.8)* | 4%; 0.0 (0.0-0.1) |
| Irregular periods | 4%; 3.7 (0.1-106.7) | 13%; 239.6 (8.2-6990.3)* | 2%; 0.2 (0.0-3.3) |
| Slow thinking | 30%; 0.5 (0.2-1.4) | 69%; 3.4 (1.4-8.4)* | 27%; 0.5 (0.1-1.4) |
| Crying | 20%; 0.6 (0.2-1.5) | 43%; 3.1 (1.2-7.7)* | 18%; 0.4 (0.1-1.5) |
| Passing more water | 22%; 0.1 (0.0-0.5) | 21%; 0.1 (0.0-0.6) | 41%; 3684.8 (12.6-1079415.0)* |
| Vomiting | 27%; 1.5 (0.2-9.3) | 19%; 0.0 (0.0-0.4) | 35%; 547.6 (2.1-141419.8)* |
| Trouble getting to clinic | 1%; 0.1 (0.0-1.5) | 4%; 0.6 (0.1-3.6) | 12% 14.9 (2.7-81.1)* |
|  | Platinum-based (N=158) | Taxane  (N=91) |  |
| Passing more water | 37%; 2.5 (1.0-6.0)* | 29%; 0.6 (0.2-1.7) |  |
| Cough | 23%; 3.2 (1.1-9.0)* | 19%; 0.8 (0.2-2.5) |  |
| Pain when swallowing | 13%; 6.3 (1.7-23.7)** | 10%; 0.7 (0.1-3.6) |  |
| Effects my social life | 50%; 2.3 (1.2-4.6)* | 46%; 0.8 (0.4-1.7) |  |
| Loss of hair | 56%; 0.4 (0.2-0.8) | 91%; 36.4 (9.9-133.7)** |  |
| General aches and pains | 32%; 1.9 (0.7-4.6) | 50%; 5.9 (1.9-17.9)** |  |
| Joint aches and pains | 27%; 0.7 (0.3-2.0) | 50%; 7.9 (2.3-27.7)** |  |
| Sore hands and feet | 16%; 3.4 (0.9-12.1) | 49%; 12.8 (2.8-58.6)** |  |
| Changes in how things smell | 25%; 0.8 (0.3-2.0) | 34%; 5.7 (1.8-19.4)** |  |
| Fever | 20%; 0.9 (0.4-2.2) | 33%; 3.4 (1.1-10.6)* |  |
| Swollen tummy | 20%; 0.7 (0.3-1.9) | 23%; 4.8 (18.1)* |  |
| Skin rash | 16%; 1.0 (0.3-2.7) | 24%; 10.8 (2.4-49.3)** |  |
|  | Females  (N=168) | Males  (N=134) |  |
| Loss of hair | 77%; 2.5 (1.1-5.6)* | 56% |  |
| Pins and needles | 45%; 2.4 (1.0-5.5)* | 33% |  |
| Nail changes | 30%; 4.6 (1.5-14.1)** | 15% |  |
| Easy bruising | 28%; 4.1 (1.5-11.6)** | 17% |  |
| Headache | 30%; 6.3 (2.1-18.2)** | 11% |  |
| Heart beating faster | 18%; 7.2 (1.7-31.1)** | 7% |  |
| Slow thinking | 53%; 2.8 (1.2-6.8)* | 25% |  |
| Excessive time spent waiting for chemo | 33%; 2.3 (1.0-5.3)* | 20% |  |
| Crying more often | 38%; 5.6 (2.2-14.2)** | 10% |  |
| Feeling unattractive | 30%; 6.4 (2.1-19.7)** | 6% |  |
| Receiving unwanted advice | 24%; 2.5 (1.1-5.8)* | 13% |  |
| Passing more water | 5 0.2 (0.1-0.6) | 7%** |  |
| Hiccups | 11 0.2 (0.1-0.5) | 24%** |  |
|  |  |  |  |
|  | Older patient (N=135) | Younger patient (N=167) |  |
| Shortness of breath | 42%; 3.4 (1.1-10.0)* | 36% |  |
| Easy bruising | 28%; 6.6 (2.1-21.2)** | 19% |  |
| Stuffy nose | 17%; 6.3 (1.5-26.7)* | 11% |  |
| Difficulty sleeping | 39%; 0.3 (0.1-0.8) | 63%* |  |
| Changes on how things smell | 19%; 0.2 (0.1-0.6) | 35%** |  |
| Painful and tender injection site | 11%; 0.1 (0.0-0.4) | 33%** |  |
| Joint stiffness | 10%; 0.1 (0.0-0.5) | 23%** |  |
| Pain when swallowing | 4%; 0.1 (0.0-0.5) | 16%** |  |
| Effects my work or home duties | 37%; 0.3 (0.2-0.7) | 60%** |  |
| Feeling angry | 10%; 0.2 (0.1-0.5) | 34%** |  |
| Seeing very unwell people | 12%; 0.3 (0.1-0.9) | 25%* |  |
|  | Chemo cycles >4 (N=105) | Chemo cycles ≤4 (N=190) |  |
| Pins and needles | 50%; 3.0 (1.4-6.5)** | 33% |  |
| Diarrhoea | 39%; 2.2 (1.0-4.5)* | 29% |  |
| Abdominal pain | 24%; 2.6 (1.1-6.4)* | 21% |  |
| Thrush in your mouth | 11%; 4.7 (1.1-19.5)* | 6% |  |
| My cancer makes me different | 32%; 2.2 (1.1-4.7)* | 22% |  |
| Indigestion | 29%; 0.4 (0.2-0.8) | 45%* |  |
| Loss of weight | 32%; 0.4 (0.2-0.9) | 41%* |  |
| Joint aches and pains | 19%; 0.2 (0.0-0.6) | 35%** |  |
| Coloured urine | 18%; 0.3 (0.1-0.8) | 30%** |  |
|  |  |  |  |
|  | Curative intent  (N=111) | Non-curative  (N=148) |  |
| Hot flushes | 39%; 2.6 (1.0-6.8)* | 23% |  |
| Changes in smell | 34%; 4.6 (1.6-13.4)* | 23% |  |
| Painful injection site | 35%; 7.3 (2.3-23.0)* | 12% |  |
| Sore eyes | 26%; 4.8 (1.4-16.7)* | 11% |  |
| Crying more often | 35%; 3.8 (1.4-9.9)* | 18% |  |
| Seeing sick people | 25%; 3.4 (1.1-10.4)* | 14% |  |
| Feeling getting treatment don’t want | 23%; 22.4 (5.0-100.1)* | 5% |  |
| Loss of appetite | 40%; 0.3 (0.1-0.8) | 53%* |  |
| Abdominal pain | 20%; 0.3 (0.1-1.0) | 25%* |  |
| Cough | 14%; 0.2 (0.1-0.8) | 25%* |  |
| Pain passing water | 3%; 0.0 (0.0-0.4) | 9%* |  |
| Cancer makes me different | 25%; 0.3 (0.1-0.9) | 28%* |  |
| No end to treatment | 14%; 0.1 (0.0-0.4) | 28%* |  |
| Not understanding what’s happening | 6%; 0.1 (0.0-0.5) | 8%* |  |

For binary demographic and treatment variables e.g. male/female and curative/non-curative only single model was estimated as estimating second model would be redundant

* p<0.05

** p<0.01

Supplementary Table 4 Percent (%) of patients placing symptom within five most severe which differentiated chemotherapy regimens and demographic groupings with odds ratio (OR) and 95% confidence interval (95% CI)

| Symptom | %; OR, (95% CI) | %; OR, (95% CI) | %; OR, (95% CI) |
| --- | --- | --- | --- |
|  | Colorectal (N=81) | Breast (N=67) | Lung (N=49) |
| Pins and needles | 15%; 8.0 (1.7-36.9)** | 0% | 4%; 0.8 (0.1-8.2) |
| Dry skin | 4%; 110.1 (2.2-5571.8)* | 1%; 0.7 (0.2-29.2) | 0% |
| Diarrhoea | 17%; 8.6 (2.0-36.1)** | 4%; 0.3 (0.0-1.8) | 4%; 0.8 (0.1-5.6) |
| Cannot concentrate | 0% | 7%; 5.6 (1.0-30.8)* | 2%; 0.5 (0.0-5.0) |
| Constant reminder of disease | 4%; 1.2 (0.3-5.3) | 7%; 5.4 (1.2-23.5)* | 2%; 0.7 (0.1-6.5) |
| Sore muscles | 0% | 0% | 12%; 42.4 (1.2-1444.5)* |
| Cough | 1%; 0.2 (0.0-20.1) | 0% | 12%; 20.9 (1.7-261.3)* |
|  | Platinum-based (N=158) | Taxane  (N=91) |  |
| General weakness | 20%; 2.7 (1.1-6.3)* | 15%; 0.8 (0.3-2.1) |  |
| Loss of appetite | 10%; 11.5 (1.7-77.9)* | 2%; 0.3 (0.1-2.0) |  |
| Pins and needles | 8%; 4.9 (1.1-23.0)* | 4%; 0.7 (0.1-3.6) |  |
| Indigestion | 7%; 6.6 (1.6-27.6)* | 2%; 0.3 (0.1-1.6) |  |
| Loss of hair | 11%; 1.1 (0.4-2.8) | 20%; 3.7 (1.3-10.0)* |  |
|  | Females  (N=168) | Males  (N=134) |  |
| Trouble swallowing | 1%; 0.0 (0.0-0.9) | 4%* |  |
|  | Older patient (N=135) | Younger patient (N=167) |  |
| General weakness | 22%; 2.6 (1.2-5.6)* | 13% |  |
| Change in the way things taste | 11%; 3.2 (1.1-9.7)* | 6% |  |
| Constipation | 14%; 2.5 (1.0-6.0)* | 8% |  |
|  | Chemo cycles>4 (N=105) | Chemo cycles≤4 (N=190) |  |
| Sore and tender muscles | 4%; 121.0 (3.0-4944.9)* | 2% |  |
| Headache | 3%; 12.7 (1.1-152.9)* | 1% |  |
|  | Curative intent  (N=111) | Non-curative  (N=148) |  |
| Loss of hair | 16%; 3.2 (1.1-9.0)* | 8% |  |
| Feeling treatment damaging | 13%; 4.1 (1.4-12.1)* | 5% |  |

For binary demographic and treatment variables e.g. male/female and curative/non-curative only single model was estimated as estimating second model would be redundant

* p<0.05

** p<0.01

Supplementary Table 5 Comparison over time in symptom severity rankings in patients with advanced disease

| Ranking | 1983 | 1993 | Current Study |
| --- | --- | --- | --- |
| 1 | Vomiting | Nausea | Affects family or partner |
| 2 | Nausea | Fatigue | Fear of future |
| 3 | Loss of hair | Loss of hair | Fatigue |
| 4 | Thought of coming for treatment | Affects family or partner | Not knowing what will happen |
| 5 | Length of time treatment takes at clinic | Vomiting | Nausea |
| 6 | Having to have a needle | Depression | Feeling my life is on hold |
| 7 | Shortness of breath | Thought of coming for treatment | Difficulty sleeping |
| 8 | Fatigue | Difficulty sleeping | Weakness |
| 9 | Difficulty sleeping | Anxiety | Constipation |
| 10 | Affects family or partner | Having to have a needle | Loss of hair |
| 11 | Affects work/home duties |  | Dependence on others |
| 12 | Trouble finding somewhere to park |  | Anxiety |
| 13 | Anxiety |  | Change in taste |
| 14 | Depression |  | Shortness of breath |
| 15 | Loss of weight |  | Feeling no end to treatment |

Ranking includes only patients with advanced/metastatic disease. Only ranks 1-10 were included in 1993 paper.
